# Evaluation of the hydrocarbon source rock and the reservoir characterization of the Minagish Formation using wireline logs

**DOI:** 10.1016/j.heliyon.2022.e09797

**Published:** 2022-06-23

**Authors:** Najeeb S. Aladwani

**Affiliations:** Department of Earth and Environmental Sciences, Faculty of Science, Kuwait University, Kuwait

**Keywords:** Middle/Oolitic Minagish reservoir, Log-derived TOC, Umm Gudair oil field, Heavy oil, Shoal depositional environment

## Abstract

We investigated the Mianagish Formation's potentiality as a Lower Cretaceous source rock and the included reservoir facies zone in the Umm Gudair oil field. The Middle Oolitic grainstone is the only producing zone in the formation. The wireline logs were used to trace the reservoir characteristics and calculate the total percentage of organic carbon (TOC) in the lithotype of the Minagish Formation. The commercial software, thin sections, and laboratory measurements are used to provide an integrated study. Integration of burial history, calculated TOC values, thermal maturity, depositional model, structural elements, and reservoir characterization were used to take a thoughtful look at the Minagish Formation's role in oil production in the field and as Lower Cretaceous source rock for the Cretaceous reservoirs. The reservoir facies are characterized by 16% average clay content, 16.7% average porosity, 420 millidarcys (mD) average permeability, and the average oil saturation is about 62%. The reservoir's quality reaches its maximum at the crest of the anticline in the west, south, and east, whereas the reservoir facies are deposited on the pre-existing structurally high shoal, while the quality decrease away from the shoal into the relatively deep water. The oil feeds the reservoir from the Lower Minagish Formation and maybe the Sulaiy Formation. According to the thermal model, the oil is heavy because of falling the TOC in the early maturation stage. The depositional environment and sequence stratigraphy are similar in the nearby Dharif and Abduliyah oil fields, and the study can be applied.

## 1 Introduction

Understanding the petroleum system elements and processes correctly in time and place lead to massive oil and gas exploration and enhances development opportunities. Also, the understanding of the reservoir characteristics is essential to evaluate the oil-in-place and producibility. In most cases, when the source rocks are heated to a particular temperature due to deep burial, they tend to release hydrocarbons that depend on the type of organic matter in the source rock (Meyer and Nederlof, 1984). The quality and quantity of organic matter in the source rock and their maturity are usually determined by chemical and microscopic analysis in the laboratory from the core samples (Zhao et al., 2016, 2017; Baouche et al., 2020; Sen and Dey, 2019). However, in most cases, the core samples are taken as a favor at certain reservoir zones, and analysis of large sets of samples may be time-consuming and costly (Zhao et al., 2016). On the other hand, the wireline logs are the most available data for the complete log in many hydrocarbon wells. Therefore, it is essential to investigate the relationship between source rocks and the wireline logs. This relationship relates to changing the physical properties of the organic matter from those of the surrounding minerals (Kamali and Mirshady, 2004). Passay et al. (1990) developed the "ΔlogR" technique to calculate the total organic carbon (TOC) in source rock using wireline logs. This technique has been calibrated and confirmed over the last decades by many such as Zheng et al. (2021), Aziz et al. (2020), Shalaby et al. (2019), Zhao et al. (2016), and Kamali and Mirshady (2004).

Kuwait's stratigraphic section has a thickness range between 23,000 and 27,000 ft and comprises three petroleum systems: The Paleozoic petroleum system, the Jurassic petroleum system, and the Cretaceous petroleum system. However, only the Jurassic and the Cretaceous petroleum systems contribute to production (Al-khamiss et al., 2009). Gotnia Formation is a thick layer of evaporites (∼200 m) which is considered a Late Jurassic seal that separates the Jurassic and Cretaceous petroleum systems (Fox and Ahlbrandt, 2002). The hydrocarbons are producing unconventionally from the Late Jurassic Sargelu, Najmah, and Marrat formations, while the Cretaceous petroleum system comprises all the petroleum system elements (Stern and Johnson, 2010; Jassim and Goff, 2006). The Upper Tithonian–Lower Berriasian Sulaiy and the Berriasian Minagish are considered the source rocks of the high potential Cretaceous reservoirs such as Zubair, Burgan, Mauddud, Wara, Ahmadi, and Mishrif formations.

**Figure 1:**
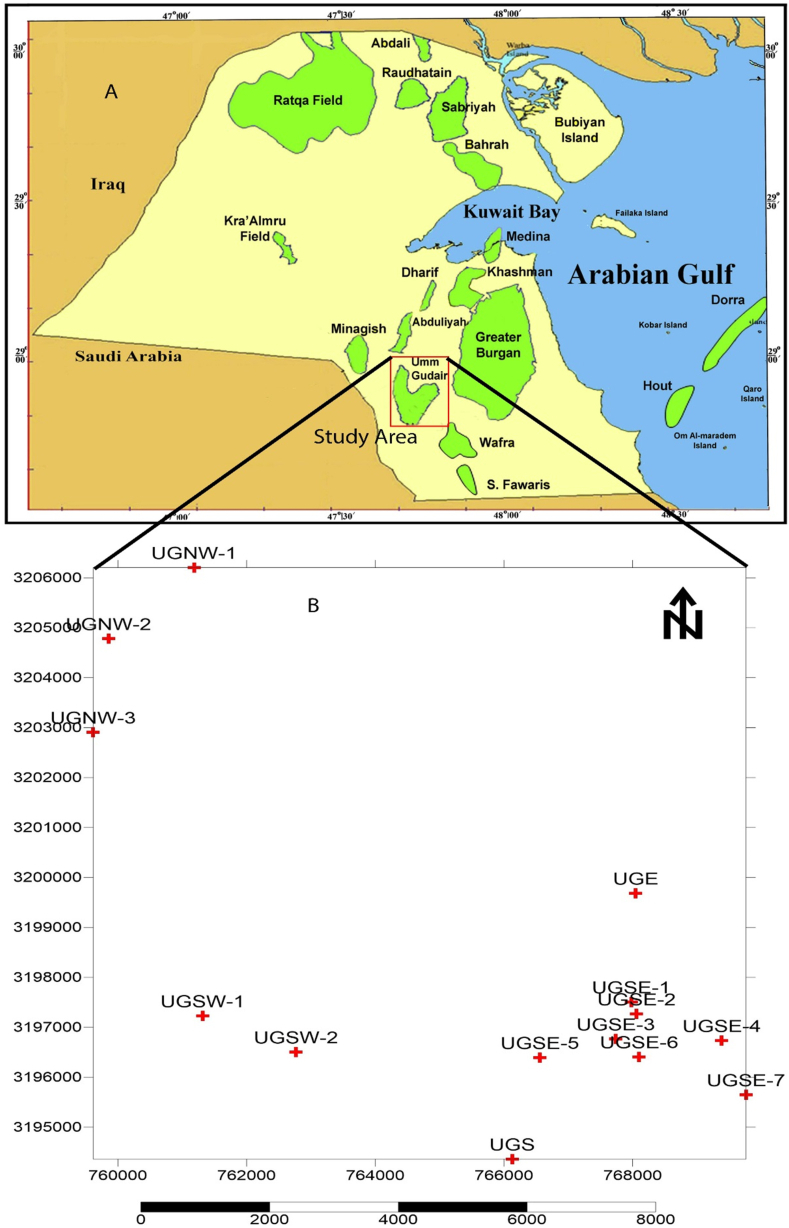
A) A) Map showing the location of the Umm Gudair oil field in Kuwait, B) Base map showing the location of the wells that have been used in this study.

**Figure 2:**
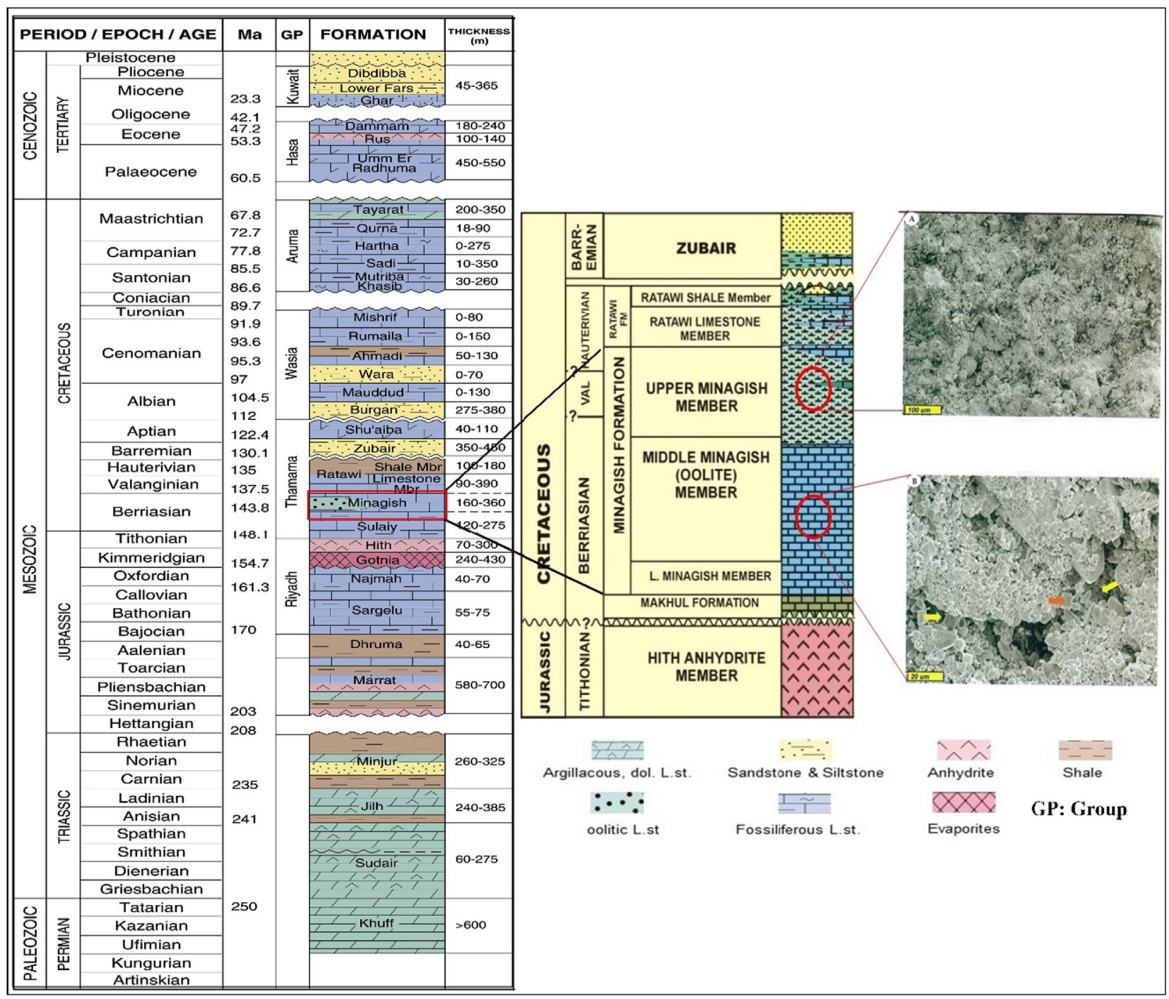
The stratigraphic column of Kuwait (after Abdulla et al., 1997), enlarged are the Lower Cretaceous Miagish and Sulaiy Formations with two thin sections A and B taken from Upper and Middle Minagish Formation; section A) Wackestone with intercrystalline or vuggy pores, occluded by microcrystalline calcite cement. B) Close-up images showing the filling of porosity by sparry calcite rhombs (yellow arrows) or occasionally halite (red arrows).

As a U-shape, the Umm Gudair Field lies in West Kuwait near the Saudi Arabian border with 172.6 km^2^ (Figure 1A). The field is divided into the western Umm Gudair sector, the eastern Umm Gudair sector, and the southern Umm Gudair sector (Figure 1B). The Minagish Oolite/Middle member of the Minagish Formation has moderate to high porosity, is hydrocarbon-bearing, and is considered the main contributor of hydrocarbon production in the Umm Gudair Field (Arasu et al., 2012). The Minagish Formation has been deposited on a broad, prograding carbonate, has a thickness range from 160 m in Burgan Field in the south to 360 m in the northern fields, and has three members. Only the Middle/Oolitic Member is a reservoir composed of fine-grained, bioturbated, and peloidal lime packstones based on the Lower Member (El Gezeery et al., 2007) (Figure 2). The upper part lithofacies is mainly peloidal wackestones-grainstones, packstone, and mudstone. The reservoir has been suggested to be heterogeneous because of the rapid decline pressure of many wells that have been tested to date, with relatively low productivity (Thomas, 2007; Zittel et al., 2008).

The above studies and many more have demonstrated the importance of the Minagish Formation as a potential source rock and reservoir. However, it is challenging to find core samples representing the complete stratigraphic units of the Minagish and Sulaiy formations. Therefore, this study aims to investigate the zones saturated by TOC and their maturity in the Minagish and Sulaiy formations using the wireline log and core samples. Another aim of this study is finding the reservoir zone and estimating its petrophysical characteristics and heterogeneities.

## 2 General geology

The Minagish Formation has been deposited on a broad, shallow intra-shelf to an inner mid-ramp environment of the passive margin of the Arabian Plate and the Neo-Tethys Ocean (Alsharhan and Naim, 1997). It is Berriasian-Valanginian in age and is divided into three members: Upper, Middle, and Lower Member. The Lower Minagish Member is a low permeability dolomitized section and dense limestone with marls, while the Upper Minagish Member comprises wackestones-grainstones and is considered the seal to the productive Minagish Oolite/Middle Member (Figure 2A) (Rahaman et al., 2012). The Minagish Oolitic is equivalent to the Ratawi Oolite of the Wafra Field and the Yamama Formation of Southern Iraq and comprises coarse-grained Oolitic grainstones and packstones with inter-crystalline or vuggy pores (Figure 2B). The Minagish Formation is overlined by the Ratawi Limestone reservoir of around 100 m and is underlain by the about 150 m Sulaiy source rock (Figure 2). The Late Jurassic and the Early Cretaceous Age's Minagish and Sulaiy formations are considered the primary source rocks of the Cretaceous reservoirs where the oil expelled to the Cretaceous reservoirs (Abdullah et al., 1997). These source rocks have kerogen Type II and were deposited in carbonate-rich, marine anoxic conditions during the Late Jurassic to Early Cretaceous time (Abeed et al., 2011). Its vitrinite reflectance (Ro) ranges between 0.52% and 0.70% and is still in the oil window after expelling 70% of the movable hydrocarbon since 60 Ma (Al-Khamiss et al., 2009).

**Figure 3:**
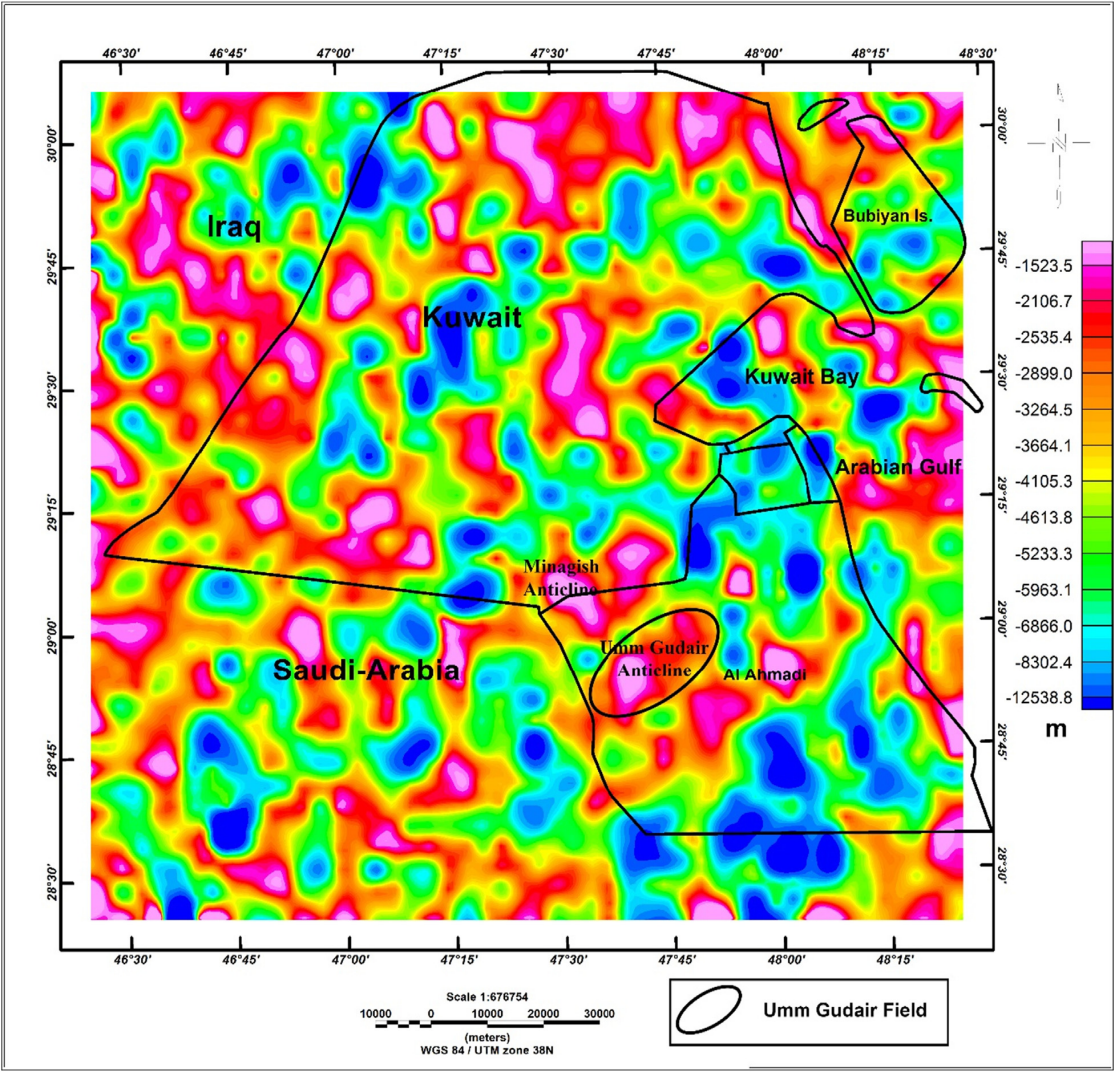
Bouguer anomalies depth map generated from Source Parameter Imaging (SPI) module in Geosoft® software shows the anticlines structure in Umm Gudair Field and nearby Minagish oil field.

The Minagish Formation is affected by Jurassic, Cretaceous, and late Tertiary structure development phases (Carmen, 1996); the main elements in Umm Gudair Field are folding and Late Cretaceous faulting. It consists of two elongated anticline structures, an east-south anticline and a west anticline, connected in a U-shape (Adasani, 1985). The Minagish Formation is affected by the tectonic events of the Cretaceous age: the break-up due to the drifting of India from Gondwanaland since 150 Ma, the subduction zone initiated from moving the Arabian Plate towards Eurasia since 130 Ma, and the ophiolite obduction onto the Arabian Margin between 80 and 90 Ma (Aladwani, 2021a; Harland et al., 1989). A set of anticlines appear on the gravity anomalies depth map (Figure 3), which is calculated by the Source Parameter Imaging (SPI) module in Geosoft® software (Aladwani, 2021b). These anticlines represent the trap in Al Ahmadi and Wafra oil field in the south of Umm Gudair oil field, Minagish and Abduliyah oil field in the north of Umm Gudair field.

## 3 Method and materials

### 3.1 Reservoir characterizations

This study used 14 wells: nine wells on the eastern anticline, three wells on the elongated western anticline, and two wells on the saddle. All wells have gamma-ray (GR), resistivity (Rt), density (ρb), neutron (ФN), and sonic (ΔT) logs. Three wells, UGSE-3, UGNW-2, and UGSE-6, reach the Lower Minagish Member and are used for Log-driving TOC of the Minagish Formation. The commercial software package (TechLog® and PetroMode®) was used to identify reservoir zones and estimated clay percentage, porosity, permeability, water saturation, and hydrocarbon saturation. Also, it is used to investigate the burial history, temperature, and thermal maturity.

We calculated the volume of shale (V_sh_) for the Oolitic grainstone of the Middle Minagish from the GR-log using Eq. (1) and confirmed the results from Neutron-Density logs.

(1) V_sh_ = 0.33 ∗ [2 (2∗IGR) – 1]

Where IGR is the index of gamma-ray, which is calculated by Eq. (2) (Schlumberger, 1974):

(2) IGR = (GR_log_ – GR_min_) / (GR_max_ – GR_min_)

Where:

GR_Log_ is the reading of the GR_log_ in the reservoir formation, GR_min_ is the minimum reading of the GRlog in front of clean sand, and GR_max_ is the maximum reading of the GR-log at shale lithology.

The porosity was calculated from the neutron and bulk density log (ρ_b_) using the Eq. (3) of Tiab and Donaldson (1996) and Eq. (4) of Wyllie et al. (1958), respectively.

(3) ФN_corr_ = ФN – (V_sh_ ∗ ФN_sh_)

Where ФN_corr_ is the corrected porosity for clean rock from shale and ФN_sh_ is the neutron porosity value for shale.

(4) ФD = (ρ_ma_ – ρ_b_) / (ρ_ma_ – ρ_f_)

Where ρ_ma_ is the density of the matrix, ρ_b_ is the bulk density measured from the log, and ρ_f_ is the fluid density.

Then, the effective porosity (Ф_e_) is calculated by the Eq. (5) of Schlumberger (1998) after correcting the total porosity from the shale effect.

(5) Ф_e_ = Ф_t_ ∗ (1 – V_sh_)

Where Ф_e_ is effective porosity, and Ф_t_ is total porosity.

The water saturation was calculated from Archie's equation (1952), equation number (6).

(6) S_w_ = [(a / Fm)∗(R_w_ / R_t_)]^(1/n)^

Where Sw is water saturation, Fm is the formation factor (=1/Ф^m^), R_w_ is derived from the value of the deep resistivity log in front of the pure water-saturated zone around the reservoir, Rt is observed deep resistivity, and (a, b, and c) are Archie's coefficients equal (1, 2, and 2, respectively).

The empirical Eq. (7) has been proposed by Wyllie and Rose (1950) and modified by Carman (1937) to calculate the permeability of the reservoirs.

(7) K = (250 × (Ф^3^/S_wir_))^2^

where K is permeability in millidarcy (mD), Ф is porosity, and S_wir_ is irreducible water saturation. The irreducible water saturation is the amount of water in the oil zone and is calculated from Eq. (8).

(8) Swir = [C/(Ф/(1 – V_cl_))]

Where V_cl_ is the volume of shale and C is Buckles's constant.

### 3.2 Petrographic analysis

Two thin sections have been taken, with a thickness range from 20 microns to 1000 microns, from core plugs in the Middle and Lower Minagish Formation to describe the grain size, sort, and packing. These thin sections were under a Scanning Electron Microscope (SEM) and showed us the types and distribution of microporosity in the carbonate (Figure 2). The JEOL SEM (Carryscope: Model JCM 5700) which characterized by 5 nm resolution has been used to carry out this analysis. The SEM can investigate the inner structure visualization using micro-CT and the microporosity distribution by providing us with three-dimensional images obtained using stereo mode.

### 3.3 Log-derived TOC and thermal maturity

**Figure 4:**
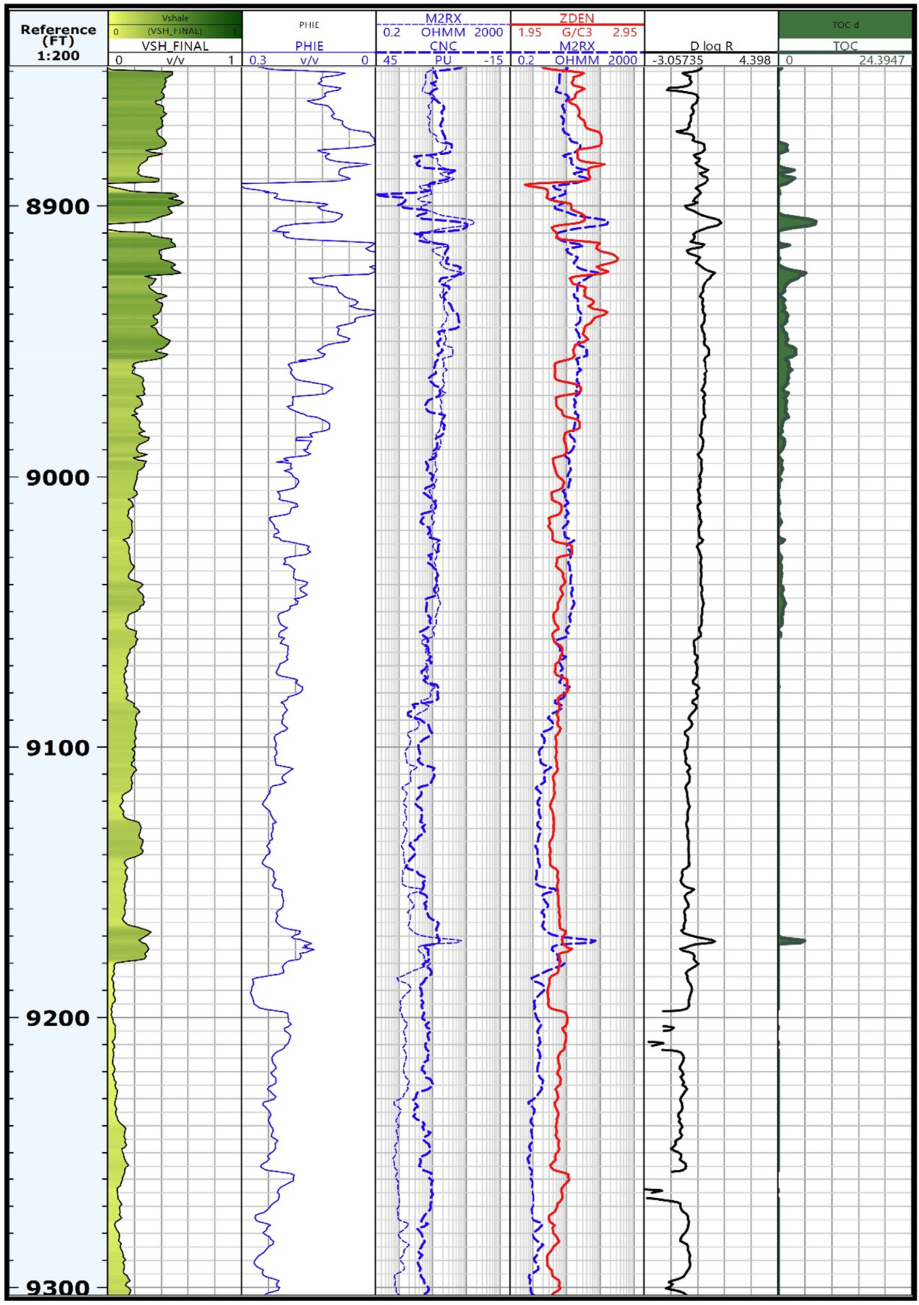
A Layout shows the log-derived TOC for well UHNW-3 using the Passey et al. (1990) method of sonic/resistivity, neutron/resistivity, and density/resistivity overlays and the calculated Δ log R separation.

This work investigated the Minagish Formation's potentiality as a source in well UGNW-3, UGNW-2, UGSE-3, and UGSE-6 in the study area penetrating the Minagish Sulaiy formations. ΔlogR technique of Passey et al. (1990) has been used to identify organic-rich intervals, including sonic/resistivity, neutron/resistivity, and density/resistivity overlays (Figure 4). This method requires an overlay of appropriately scaled sonic, neutron, and density logs on a resistivity curve using some empirical Eqs. (9, 10, and 11):

(9) Δ log R (sonic) = log10 (R/R _baseline_) + 0.02 × (Δt – Δt _baseline_)

(10) Δ log R (neutron) = log10 (R/R _baseline_) + 4.00 × (ՓN – ՓN _baseline_)

(11) Δ log R (density) = log10 (R/R _baseline_) – 2.50 × (ρb – ρb _baseline_)

Where Δ log R is the curve separation measured in the logarithmic resistivity cycle. The value of Δ log R is calculated essentially from sonic/resistivity logs, confirming the results from neutron/resistivity and density/resistivity logs. The separation value “Δ log R” at a specific zone reflects the saturation of that zone by organic matters.

R is the resistivity (ohm-m) measured by the resistivity logging tool. The ADR™ Azimuthal Deep Resistivity tool developed by Halliburton Company has been used in well logging.

R _baseline_ is the resistivity corresponding to the Δt _baseline_.

ՓN baseline and ρb _baseline_ are curve values for non-source rock lithology, clay-rich rocks.

Δt is the measured transit-time in msec/ft.

ՓN is the measured neutron porosity scaled in the fraction unit.

ρ_b_ is the measured bulk density in g/cm^3^.

The Δ Log R separation is linearly related to the TOC and is a function of maturity. The TOC in organic-rich rocks can be derived from Δ Log R by the Eq. (12):

(12) TOC = (Δ log R) × 10 ^(2.297 – 0.1688 × LOM)^

LOM (Level of Organic Metamorphism) is estimated from the burial history, thermal history, and the cross-plot between the maximum temperature (T_max_) and effective heating time (T_eff_) (Diab and Khalil, 2021; Mohamed et al., 2016; Hood et al., 1975; Passey et al., 1990). First, the burial history model was built using the Petromode® software, and then the LOM was derived from it by plotting the relationship between the effective heating time (T_eff_) versus the maximum temperature (T_max_). This model has been calibrated by the vitrinite reflectance (R_o_) model developed by Burnham et al. (2017).

## 4 Results

### 4.1 Thermal maturity hydrocarbon potentiality

**Figure 5:**
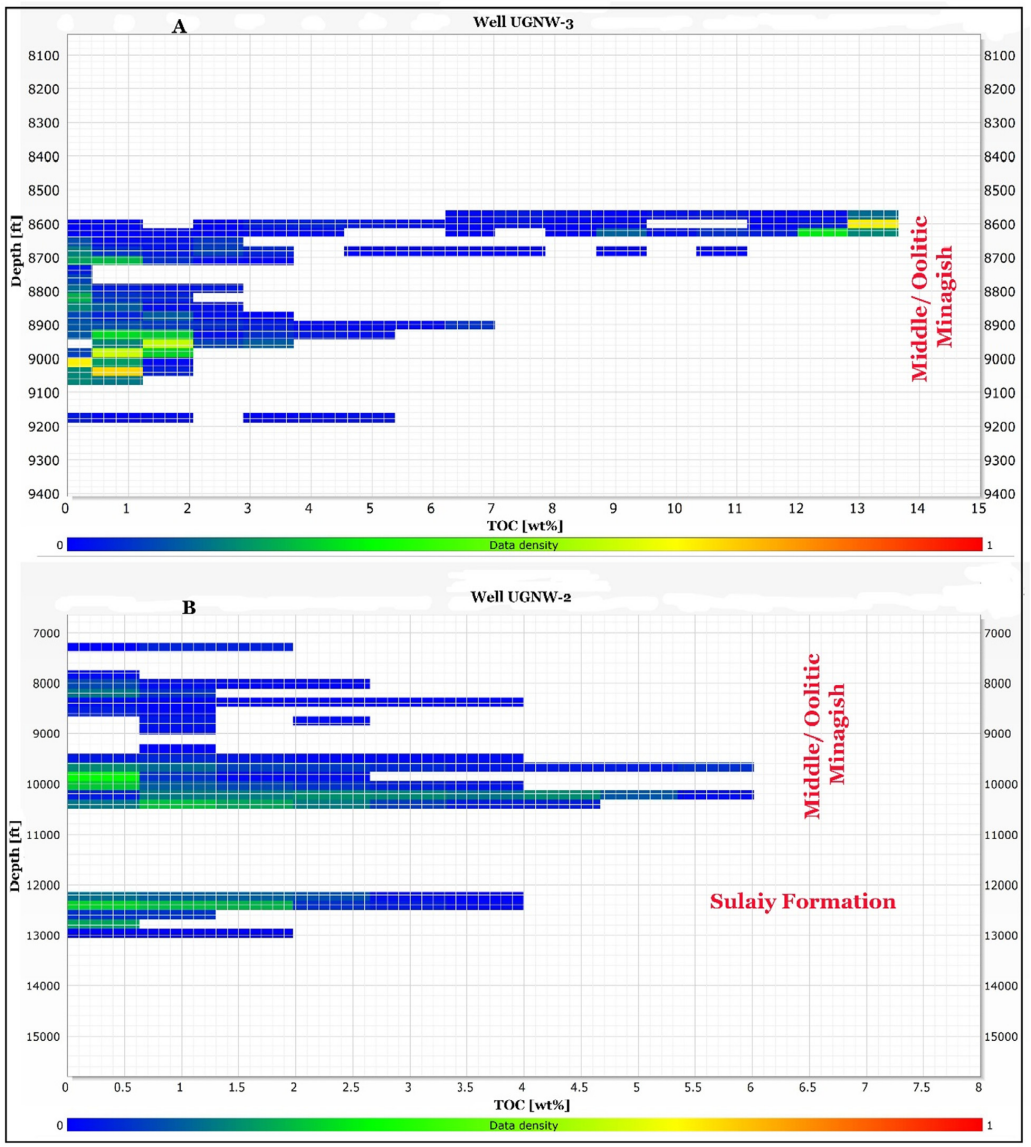
Cross-plot between the log-derived TOC and its depth in A) well UGNW-3 and B) well UGNW-2, showing that the values of the TOC range from less than 1 up to 6.9 wt%, while the total organic matters concentrated mainly in two depth ranges (8,500–10,500 ft) and (12,200–13,000 ft).

**Figure 6:**
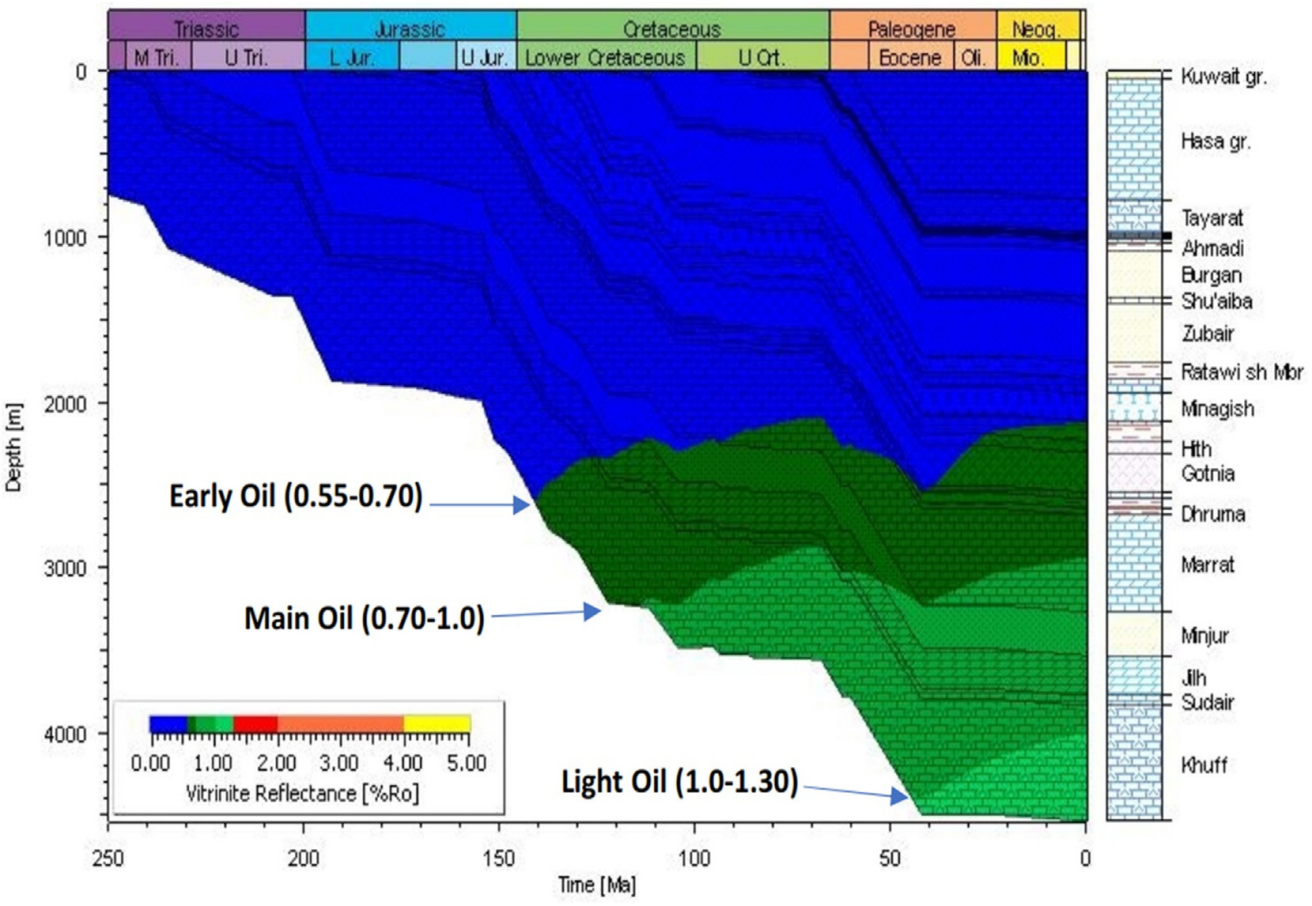
The burial history of Umm Gudair field derived from the depositional sequence in the field, calibrated by the vitrinite reflectance (R_o_), showing that the Minagish Formation lies in the early stages stage of the oil window.

We investigated the source rock potentiality of the Minagish Formation rocks and the underlying Sulaiy formations from the deep wells at the eastern and southern anticlines (UGSE-3 and UGSE-6) and the western anticline (UGNW-2 and UGNW-3). The deep wells' depth reaches 15,750 ft and penetrates the Lower Minagish and Sulaiy Members. The log-derived TOC from well UGNW-3 (Figure 5A) shows high correlation values with the laboratory measurement TOC (Table 1) for Oolitic Minagish Formation. The well UGNW-2 penetrates the anhydrite of the Hith Formation at 13,000 ft depth, while the Minagish Oolitic reservoir depth ranges between 8,000 and 10,750 ft. The log-derived TOC values (Figure 5B) range between less than 1 wt.% and 6.9 wt.% in the Middle Minagish Member; however, the most values condensed down to 2 wt.% (Figure 5B). The LOM of the Minagish Formation is 9.2, while it is 9.8 for the Sulaiy Formation, which indicates that these formations have entered the oil maturation zone. The thermal maturity model shows that the R_o_ of the Minagish Formation ranges between 0.55% and 0.70%, whereas it lies at the beginning of the heavy oil window (Figure 6). Abdullah and El Gezeery (2016) measured the TOC in core samples from the Minagish Formation in the Umm Gudair field and found that the TOC ranges between 0.99 and 7.9 wt.%. Also, they found that T_max_ does not exceed 423 °C, which indicates immature kerogen or at the early stage of maturation. The log-derived TOC values are well correlated to those measured from the samples and advanced by its deduction from the well logs and coverage of the complete logged sedimentary succession, which ranges between 7,000 and 15,500 ft.

**Table 1:** The results of measured TOC from the studied sample in well UG-NW-3.

Well Name	Sample type	Formation	Samples Depth (ft)	TOC (Avg.) (wt%) Measured	TOC (wt%) Log-drived	Tmax	Ro (%) Cal_Tmax	Ro (%) Cal_1D Model	
UG-NW-3	Cutting	Minagish	8520–8540	1.08		433	0.63	0.55–0.70	Early oil
UG-NW-3	Cutting	Minagish	8700–8720	0.85	1.05 (0.61–1.41)	434	0.65	0.55–0.70	
UG-NW-3	Cutting	Minagish	8930–8940	1.02	1.060 (.47–1.69)	430	0.58	0.55–0.70	
UG-NW-3	Cutting	Minagish	9170–9180	1.51	2.15 (0.7–3.6)	432	0.62	0.55–0.70

### 4.2 Depositional environment

**Figure 7:**
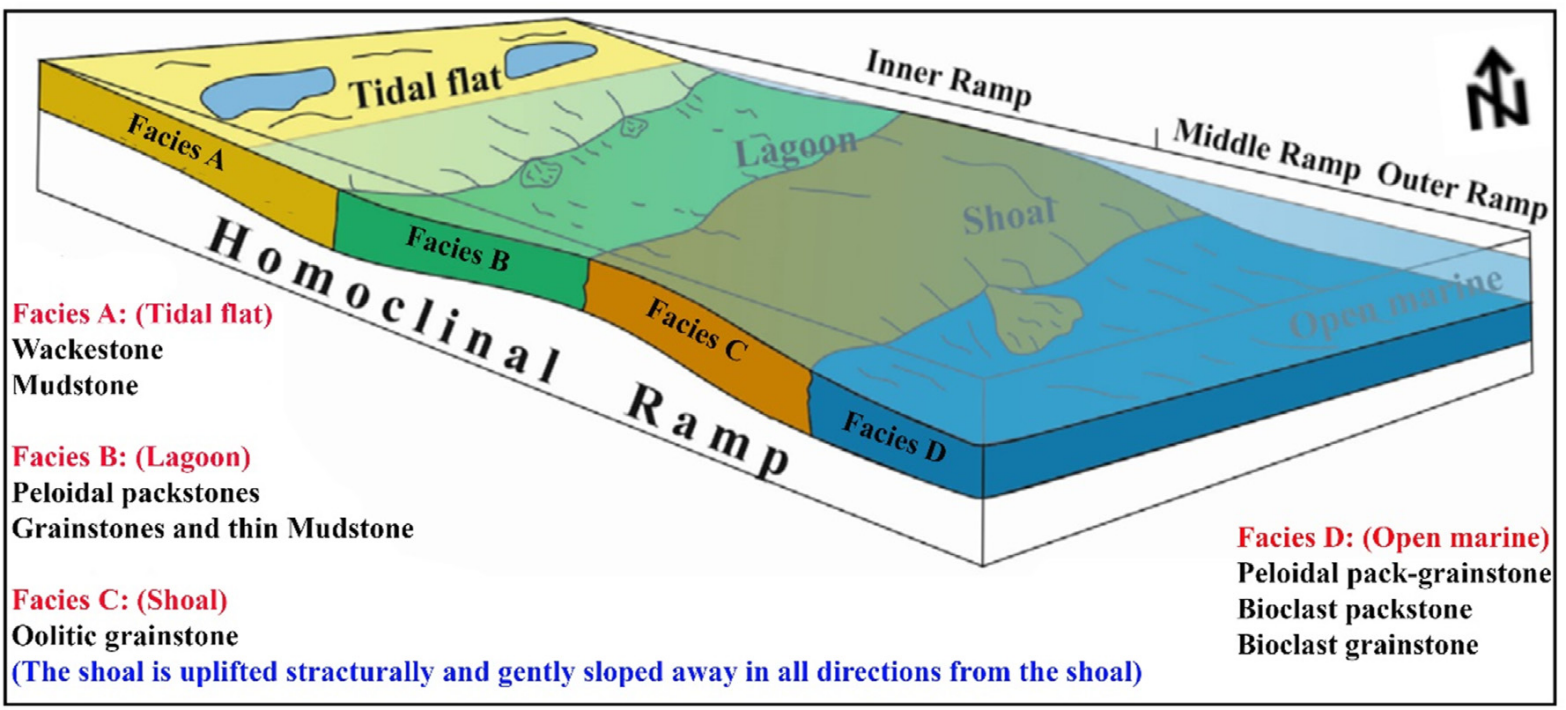
The depositional model of the Minagish Formation in the study area represents a homoclinal ramp extended from the inner ramp to open marine, based on the facies heterogeneity and structural effect of the area (modified from Aghaei et al., 2013).

The Minagish Formation is deposited in a shallow marine environment on the inner to middle carbonate ramp with a dominant limestone lithology and a wide range of lithofacies from mudstone to grainstone. The reservoir facies is developed in the formation's Middle/Oolitic Member. It is composed of fine to coarse-grained Oolitic grainstones and packstones with bioclasts. It is interpreted to have been deposited on a pre-existing structural high shoal. This shoal is high and gently sloped away into a deeper environment in all directions from the shoal (Figure 7). The dominant lithotype in the lower part of the Minagish Formation is grainstone, while packstone, wackestone, and mudstone are developed in the upper part (Figure 7) (Datta et al., 2013).

### 4.3 Sequence stratigraphy

**Figure 8:**
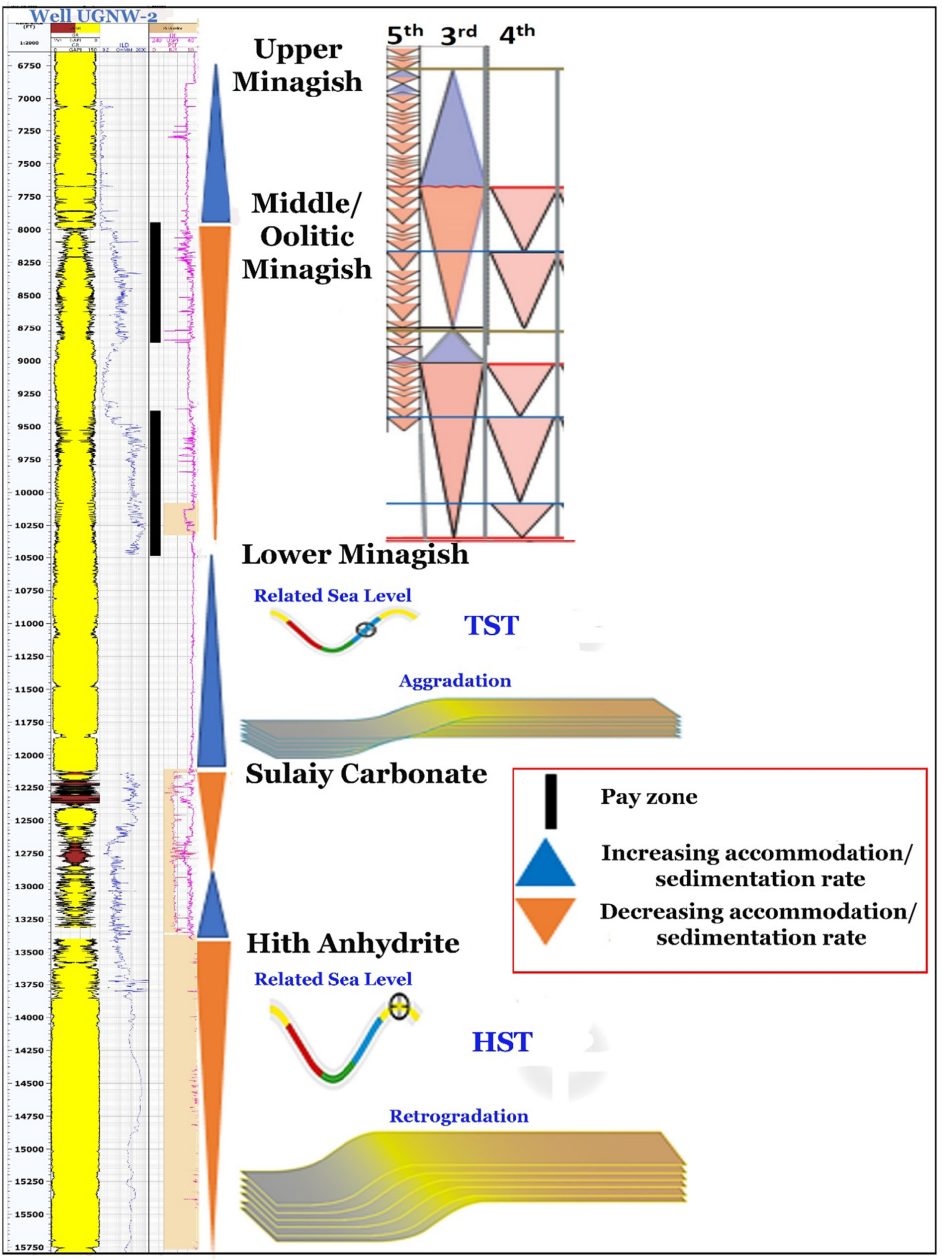
Sequence stratigraphy for the deep well (UGNW-2) that penetrate the Minagish, Sulaiy, and Hith Anhydrite Formations.

**Figure 9:**
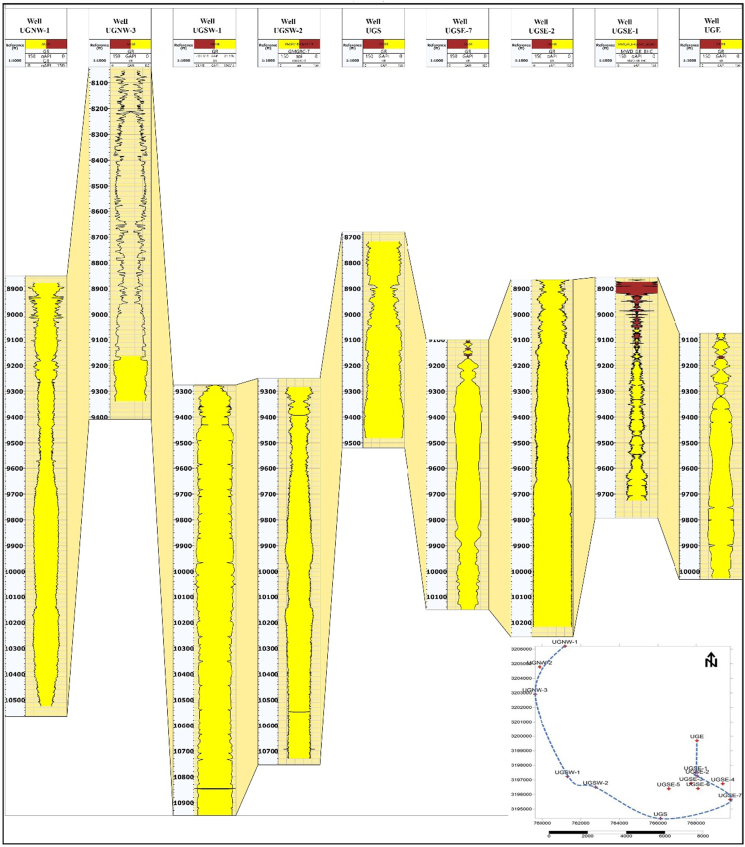
Wells correlation chart for the wells UGNW-1, UGNW-3, UGSW-1, UGSW-2, UGS, UGSE-7, UGSE-2, UGSE-1, and UGE, was ordered from the northern west and passing through the two anticline arms in the west, the south, and the east.

There is a sharp contact which appears on the GR-log between the peloidal wackestone to packstone of the Upper Minagish Member, which is characterized by a Transgressive Systems Tract (TST), and the medium- to coarse-oolitic grainstone of the Middle Minagish member, which is characterized by a Highstand Systems Tract (HST) (Nath et al., 2014; Datta et al., 2013) (Figure 8). The Middle Oolitic grainstone is the producing reservoir deposited in a single third-order sea-level cycle and is composed of stacked broadly coarsening parasequences upward due to a high prograding ramp setting (Nagm et al., 2018; Wolpert et al., 2015) (Figure 8). There is strong evidence from the GR-log about the changing of the reservoir facies into more argillaceous limestone toward the north of the study area, indicated by the shape and magnitude of the recorded GR (Figure 9). This lateral heterogeneity may be due to changing the water depth laterally into deep water away from the shoal.

### 4.4 Reservoir characterizations

**Figure 10:**
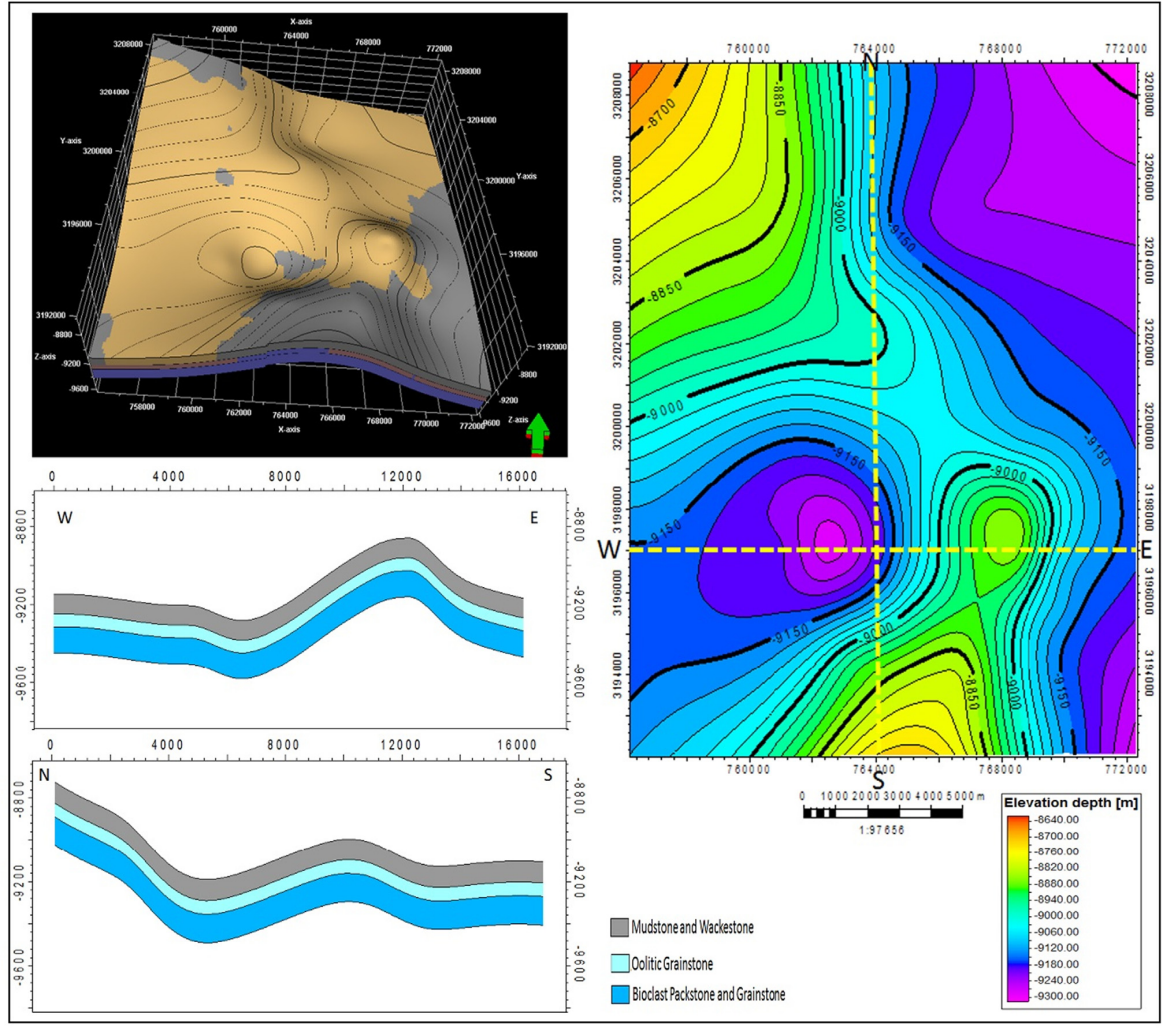
The structural elements that affect the Umm Gudair oil field, obtained through structure contour map at the right and its 3D model in the upper left, with two geologic cross-sections: North-South and East-West showing the anticlinal structure in the field.

**Figure 11:**
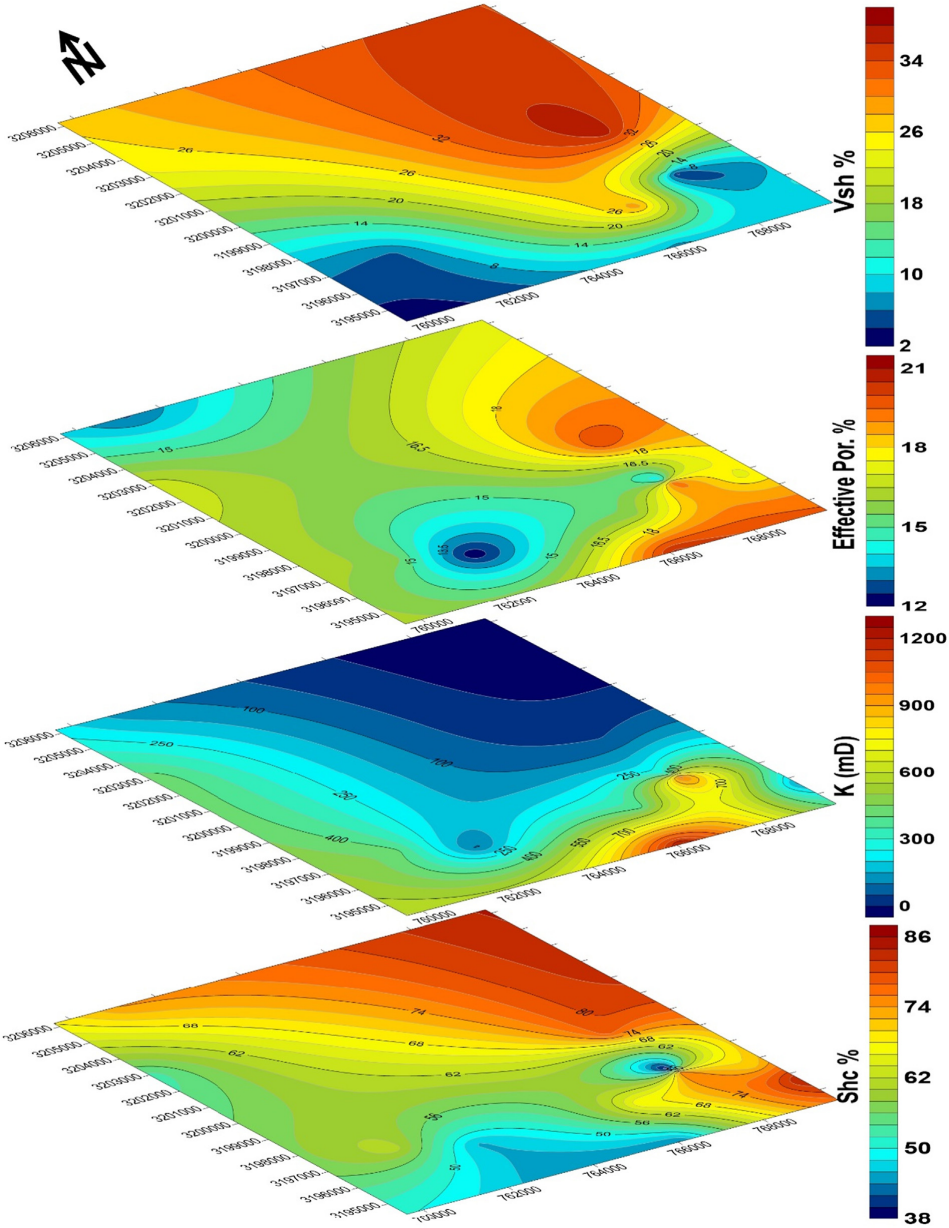
Petrophysical parameters maps showing the distribution of shale volume (V_sh_ in %), effective porosity (ɸ_eff_ in %), permeability (K in millidarcy, mD), and hydrocarbon saturation (S_hc_ in %) in the Umm Gudair oil field.

Only Minagish Middle/Oolitic grainstone is the reservoir facies in the Minagish Formation. The reservoir lies at a depth range between 8040 and 9250 ft and is characterized by an overall domal structure in the Umm Gudair oil field. Two elongated anticlines having N-NW direction and NE direction (Figures 9 and 10) represent the domed structure. A shallow saddle separates the two anticlines, and each accumulates the oil in the crest. The reservoir has a tarmat 50–100ft thick at the base of the oil column (El Gezeery et al., 2008; Abdullah et al., 2005). The reservoir quality is affected by cementation and compaction, whereas the porosity and permeability depend on the leaching of the carbonate grains. The average clay content is 16%, increasing to 30% in the northeast of the field away from the western and southeastern anticlines, whereas the reservoir facies are deposited on a structurally high shoal (Figure 11). The porosity ranges between 12 and 20%, with an average of 16.7%, while the average permeability is 420 millidarcy (mD) and increases at the anticlines' crest to 1200 mD. The hydrocarbon saturation ranges from 37% to 65% in the anticline crests in the west, the south, and the southeast (Figure 11).

## 5 Discussion

When the structure map of the Minagish Formation is compared to the historical tectonic events of Kuwait, then the anticlines of Umm Gudair, Dharif and Abduliyah areas are interpreted to be related to a regional West Kuwait Arch Lineament called the intra-Jurassic level structures (Carman, 1996). During the Berriasian age, Kuwait was exposed to regional flooding (Rahaman et al., 2012) and became a homoclinic carbonate ramp (Figure 7). Hence, the Minagish Formation was deposited on the pre-existing anticlines as a shoal environment in all these fields. After integrating the depositional environment (Figure 7), wells correlation along with the trap (Figure 9), the structural elements of the Umm Gudair field (Figure 10) with the petrophysical parameters maps (Figure 11), it has been found that the reservoir characterized by lateral heterogeneity. The leading cause of the heterogeneity is related to the structure that affects the shoal. The best reservoir facies quality lies in the anticline area in the west, south, and southeast that was deposited on the shoal. Its quality decreases away from the shoal in all directions, whereas the environment becomes deeper and the clay percent increases, consequently changing the porosity and permeability. Consequently, for the best development plan for the field, we should avoid drilling far away from the crest of the anticlines axis that extends as a U-shape appeared in Figures 1 and 3, 9, 10, and 11.

The "ΔlogR" technique estimated the TOC with high accuracy compared to the core samples analysis in the complete lithotype of the Minagish Formation. It is found that the TOC values are concentrated in the Middle Minagish Member and the Sulaiy Formation. The results from this study and others have demonstrated how well logs are related to source rocks. For example, Kamali and Mirshady (2004) quantified the correlation between wireline logs (sonic, density, neutron, and resistivity logs) and total organic carbon using the "ΔLogR" calculation approach. Also, Aziz et al. (2020) estimated TOC using different logs relationship, including the method of "ΔLogR" and found that it is a high accuracy method for identifying the TOC from the wireline logs. In this study, the TOC values are highly comparable with the previous study carried out in this field. However, this study determined the complete zones of high TOC content that otherwise would be difficult to study the complete section through core sampling. In Umm Gudair Field, the hydrocarbon migrates from the rich-TOC member of the Minagish Formation and the underlying Sulaiy Formation to the Middle/Oolitic grainstone Member and is capped by the Upper Member, which composes of wackestone and mudstone.

## 6 Conclusion

In this study, we have tried to provide an understandable scenario of the oil generation, maturity, and production for the Lower Cretaceous Minagish Formation through the following:

1 Deriving the TOC values for the Minagish and Sulaiy Formations using the “ΔLog R″ separation technique from overlaying the sonic/resistivity, neutron/resistivity, and density/resistivity logs of deep wells in the Umm Gudair Field.
2 The log-derived TOC values range from less than 1 wt% and up to 6.9 wt% in depth ranges (8,500–10,500 ft) and (12,200–13,000 ft).
3 The calculated thermal maturity for the Minagish and Sulaiy formations coincides with the measured values and indicates that the total organic matter is still in the early stage of maturation (heavy oil), which led to the tarmat in the base of the oil column (50–100 ft) in the trap.
4 The reservoir facies are the Middle/Oolitic grainstone and has been investigated structurally, petrophysically, and using thin sections. It has been found that the dominant structure is domal and consists of two anticlines elongated in the northwest and the southeast directions.
5 The reservoir is characterized by high quality, especially at the crests of the anticlines, whereas the porosity reaches 20%, permeability 1200 mD, and oil saturation reaches 65%.
6 The lateral heterogeneity characterizes the reservoir due to the deposition of the reservoir facies on a height shoal.

## Declarations

### Author contribution statement

Najeeb S. Aladwani: Conceived and designed the experiments; Performed the experiments; Analyzed and interpreted the data; Contributed reagents, materials, analysis tools or data; Wrote the paper.

### Funding statement

This research did not receive any specific grant from funding agencies in the public, commercial, or not-for-profit sectors.

### Data availability statement

The data that has been used is confidential.

### Declaration of interest's statement

The authors declare no conflict of interest.

### Additional information

No additional information is available for this paper.

## References

[bib1] Abdullah F.H., El Gezeery T. (2016). Organic geochemical evaluation of hydrocarbons in lower cretaceous middle minagish reservoir, Kuwait. Mar. Petrol. Geol..

[bib2] Abdullah F.H., Carpentier B., Kowalewski I., Van Buchem F., Huc A.Y. (2005). Organic matter identification in source and reservoir carbonate in the Lower Cretaceous Mauddud Formation in Kuwait. GeoArabia.

[bib3] Abdullah F.H.A., Nederlof P.J.R., Ormerod M.P., Kinghorn R.R.F. (1997). Thermal history of the lower and middle cretaceous source rocks in Kuwait. GeoArabia.

[bib4] Abeed Q., Al-khafaji A.J., Littke R. (2011). Source rock potential of the upper Jurassic lower cretaceous succession in the southern part of the mesopotamian basin (Zubair subzone), southern Iraq. J. Petrol. Geol..

[bib5] Adasani M. (1985).

[bib6] Aghaei A., Mahboubi A., Moussavi-Harami R., Heubeck C., Nadjafi M. (2013). Facies analysis and sequence stratigraphy of an upper Jurassic carbonate ramp in the eastern Alborz range and Binalud Mountains, NE Iran. Facies.

[bib7] Aladwani N.S. (2021). Assessment of petroleum system of arabian-Iranian basin in Kuwait. All Earth.

[bib8] Aladwani N. (2021). Qualitative and quantitative analysis of the crustal basement structure using gravity anomaly maps of Kuwait. Kuwait J. Sci..

[bib9] Al-Khamiss A., AbdulMalik S., Hameed W.A. (2009). EAGE Workshop on Detective Stories behind Prospect Generation-Challenges and the Way Forward.

[bib10] Alsharhan A.S., Nairn A.E.M. (1997). Sedimentary Basins and Petroleum Geology of the Middle East.

[bib11] Arasu R.T., Nath P.K., Khan B., Ebrahim M., Rahaman M., Bader S., Abu-Ghneej A.F.N. (2012). SEG Technical Program Expanded Abstracts 2012.

[bib12] Archie G.E. (1952). Classification of carbonate reservoir rocks and petrophysical considerations. AAPG (Am. Assoc. Pet. Geol.) Bull..

[bib13] Aziz H., Ehsan M., Ali A., Khan H.K., Khan A. (2020). Hydrocarbon source rock evaluation and quantification of organic richness from correlation of well logs and geochemical data: a case study from the sembar formation, Southern Indus Basin, Pakistan. J. Nat. Gas Sci. Eng..

[bib14] Baouche R., Sen S., Debiane K., Ganguli S.S. (2020). Integrated reservoir characterization of the Paleozoic and Mesozoic sandstones of the El ouar field, Algeria. J. Petrol. Sci. Eng..

[bib15] Burnham A.K., Peters K.E., Schenk O. (2017).

[bib16] Carman G.J. (1996). Structural elements of Onshore Kuwait. GeoArabia: Middle East Petrol. Geosci..

[bib17] Carman P.C. (1937). Fluid flow through granular beds. Trans. Inst. Chem. Eng..

[bib18] Datta K., Ghosh D., Al-Nasheet A., Clark W., Yaser M., Ma Z., Gomez E., Bond D. (2013 June). 75th EAGE Conference & Exhibition Incorporating SPE EUROPEC 2013 (cp-348).

[bib19] Diab A.I., Khalil H.M. (2021). Quantitative assessment of the tight gas reservoirs in the Obaiyed field, Shushan Basin, NW Egypt. NRIAG J. Astronom. Geophys..

[bib20] El Gezeery T., Al-Qabandi S., Ebaid A. (2008). GEO 2008 middle east, Geoscience Conference & Exhibition, 3e5 March 2008.

[bib21] El-Gezeery T., Conradi C., Zereik R., Pearce T., Dix M. (2007. November). American Association of Petroleum Geologists.

[bib22] Fox J.E., Ahlbrandt T.S. (2002). U.S. Geological Survey Bulletin E.

[bib23] Harland W.B., Armstrong R.L., Cox A.V., Craig L.E., Smith A.G., Smith D.G. (1989).

[bib24] Hood A., Gutjahr C.C.M., Heacock R.L. (1975). Organic metamorphism and the generation of petroleum. AAPG (Am. Assoc. Pet. Geol.) Bull..

[bib25] Jassim S.Z., Goff J.C. (2006).

[bib26] Kamali M.R., Mirshady A.A. (2004). Total organic carbon content determined from well logs using ΔLogR and Neuro Fuzzy techniques. J. Petrol. Sci. Eng..

[bib27] Meyer B., Nederlof M. (1984). Identification of source rocks on wireline logs by density/resistivity and sonic transit time/resistivity cross plots. AAPG (Am. Assoc. Pet. Geol.) Bull..

[bib28] Mohamed A.Y., Whiteman A.J., Archer S.G., Bowden S.A. (2016). Thermal modeling of the Melut basin Sudan and South Sudan: implications for hydrocarbon generation and migration. Mar. Petrol. Geol..

[bib29] Nagm E., Bamousa A., Memesh A., Babikir I.A., Dini S. (2018). Relative sea-level changes and sedimentary facies development of the lowermost Cretaceous (Berriasian–Valanginian) cycles in the north of Ar Riyad city, Saudi Arabia. J. Asian Earth Sci..

[bib30] Nath P.K., Singh S.K., Ye L., Al-Ajmi A.S., Bhukta S.K., Al-Otaibi A.H. (2014 January). International Petroleum Technology Conference.

[bib31] Passey Q.R., Creaney S., Kulla J.B., Moretti F.J., Stroud J.D. (1990). A practical model for organic richness from porosity and resistivity logs. AAPG (Am. Assoc. Pet. Geol.) Bull..

[bib32] Rahaman M., Ebrahim M., Al Zuabi Y., Gandhi D. (2012). GEO 2012 Middle East, Geoscience Conference & Exhibition.

[bib33] Sen S., Dey J. (2019). A field-scale overview of facies architectures and depositional environment integrating core and geophysical log data: study from a marginal Gondwana Basin, India. J. Geol. Soc. India.

[bib34] Shalaby M.R., Jumat N., Lai D., Malik O. (2019). Integrated TOC prediction and source rock characterization using machine learning, well logs and geochemical analysis: case study from the Jurassic source rocks in Shams Field, NW Desert, Egypt. J. Petrol. Sci. Eng..

[bib35] Schlumberger (1998).

[bib36] Schlumberger (1974).

[bib37] Stern R.J., Johnson P. (2010). Continental lithosphere of the arabian plate; a geologic, petrologic, and geophysical synthesis. Earth Sci. Rev..

[bib38] Tiab D., Donaldson E.C. (1996).

[bib39] Thomas O. (2007).

[bib40] Wolpert P., Bartenbach M., Suess P., Rausch R., Aigner T., Le Nindre Y.M. (2015). Facies analysis and sequence stratigraphy of the uppermost Jurassic–lower cretaceous Sulaiy formation in outcrops of central Saudi Arabia. GeoArabia.

[bib41] Wyllie M.R.J., Gregory A.R., Gardner G.H. (1958). An experimental investigation of the factors affecting elastic wave velocities in porous media. Geophysics.

[bib42] Wyllie M.R.J., Rose W.D. (1950).

[bib43] Zhao W., Li S., Yao H., Zhang S., Zhang Y., Yang B., Hou J. (2017). Molecular optimization enables over 13% efficiency in organic solar cells. J. Am. Chem. Soc..

[bib44] Zhao L., Qiu G., Anderson C.W., Meng B., Wang D., Shang L., Yan H., Feng X. (2016). Mercury methylation in rice paddies and its possible controlling factors in the Hg mining area, Guizhou province, Southwest China. Environ. Pollut..

[bib45] Zheng D., Wu S., Hou M. (2021). Fully connected deep network: an improved method to predict TOC of shale reservoirs from well logs. Mar. Petrol. Geol..

[bib46] Zittel R.J., Beliveau D., O'Sullivan T., Mohanty R., Miles J. (2008). Reservoir crude oil viscosity estimation from wireline NMR measurements-Rajasthan, India. SPE Reservoir Eval. Eng..

